# Exploring anticipated barriers to engagement with Martha’s Rule, a patient/family-activated escalation system to support early detection of patient deterioration, among ethnically and culturally diverse groups: a qualitative study

**DOI:** 10.1136/bmjopen-2026-123741

**Published:** 2026-11-09

**Authors:** Eleanor Lutman-White, Olufunmilola Christiana Ladeinde, Liz Lees-Deutsch, Rachel Chapman

**Affiliations:** 1 Research Centre for Healthcare and Community Transformation, Coventry University, Coventry, UK; 2 Centre for Care Excellence, University Hospitals Coventry and Warwickshire NHS Trust, Coventry, UK; 3 University Hospitals Coventry and Warwickshire NHS Trust, Coventry, UK; 4 Coventry City Council, Coventry, UK

**Keywords:** Health Services Accessibility, Safety, QUALITATIVE RESEARCH

## Abstract

**Abstract:**

**Objectives:**

This study aimed to increase understanding of the barriers faced by those from diverse ethnic and cultural backgrounds in engaging with Martha’s Rule—a patient/family-activated escalation system to support early detection of patient deterioration.

**Design:**

Qualitative study using semistructured in-depth interviews conducted between June and September 2025. Data was analysed using Framework Approach and Leveque’s conceptual framework of healthcare access.

**Setting:**

An acute hospital Trust in England.

**Participants:**

22 community members from ethnically and culturally diverse backgrounds.

**Results:**

Participant narratives highlight multiple potential barriers to accessing Martha’s Rule for this population at the system and patient level. The findings demonstrate the importance of ensuring the visibility and accessibility of information about Martha’s Rule. Socio-cultural factors affecting ability to access Martha’s Rule included language difficulties and perceived lack of agency that underpinned a reluctance to raise concerns. Poor previous experiences of healthcare and anticipated negative responses from staff are key factors that reduce patients’ trust and confidence, potentially impacting on engagement with Martha’s Rule.

**Conclusions:**

This qualitative study has illuminated the individual and system-level barriers to accessing Martha’s Rule. Recommendations based on the findings include circulating information about Martha’s Rule widely, including through community outreach and taking into consideration patient/family members’ language abilities and digital skills, increasing staff awareness that cultural norms may discourage questioning authority, supporting a culture of speaking up, building trust in Martha’s Rule and addressing distrust and uncertainty in healthcare.

**STRENGTHS AND LIMITATIONS OF THIS STUDY**

- This study generated rich insights into the potential barriers and enablers to engagement with Martha’s Rule, a patient/family-activated escalation system implemented in England, for patients and families from diverse ethnic and cultural backgrounds.
- Provision of interpreters enabled inclusion of the perspectives from participants who did not speak English.
- Recommendations based on the findings can be used by National Health Service healthcare trusts to enhance equitable access to Martha’s Rule.
- Participants had no direct experience of Martha’s Rule as it was at the early stages of implementation when the interviews took place and therefore our findings reflect anticipated rather than experienced barriers and enablers.

## Introduction

 Martha Mills died aged 13 in 2021 after failures to identify and properly treat her sepsis that developed while she was in hospital. Martha’s family’s concerns about her deteriorating condition were not responded to and in 2023 a coroner ruled that Martha would probably have survived had she been moved to intensive care earlier.

In response to this and other cases related to the management of patient deterioration, the National Health Service (NHS) has implemented patient/family-activated escalation systems across all acute Trusts in England (known as Martha’s Rule) from April 2024.^1^ Such systems enable patients and family members to alert critical care outreach or medical emergency teams directly when they have concerns about a condition which is worsening, specifically when concerns are not responded to in a timely manner. With the aim of preventing avoidable harm, a key component of implementation is a 24-hour phone line that patients and family members can use if their concerns about changes in the condition of a relative/loved one are not being responded to by clinical staff in the ward area.

However, patients and family members may experience barriers to raising their concerns and using patient-led escalation systems. Evidence suggests that lack of awareness, power imbalances between patients/relatives and healthcare staff, communication challenges, cultural, spiritual and personal beliefs and fear of impact on patient-staff relationships are all potential barriers to patients and family members engaging with patient-led escalation systems.^2–8^ It is likely that those from diverse ethnic and cultural backgrounds will experience these barriers more acutely as there is evidence that suggests that ethnic minority groups may experience greater challenges accessing healthcare,^9–14^ have poorer experiences of healthcare,^15 16^ experience language barriers and have lower levels of health literacy^17^ which may impact on willingness and ability to raise concerns and engage with patient-led escalation systems. The NHS Race and Health Observatory suggest that ‘Introducing patient safety measures such as Martha’s Rule may only serve to give greater agency to those who already have it within the system and fail to protect the most marginalised in society’.^18^ Examination of the impact of diverse ethnic and cultural backgrounds on willingness and ability to engage with patient-led escalation systems is lacking from the research evidence base.^8^ Patients from diverse ethnic and cultural backgrounds are potentially more likely to have greater need to use a patient/family activated system as there is evidence that patients from ethnic minority backgrounds are more likely to experience avoidable harm in healthcare^19^ and are at increased risk of dying from sepsis.^20^ Research is therefore required to understand whether Martha’s Rule is likely to meet the needs of patients from diverse ethnic and cultural backgrounds.

This study sought to better understand how population groups with diverse ethnic and cultural backgrounds might engage with a patient-led escalation system and the barriers faced in relation to engagement.

## Methods

### Study design

This was a qualitative study comprising interviews with community members from ethnically and culturally diverse backgrounds. Methods and results are reported according to the Consolidated Criteria for Reporting Qualitative Research guidelines.^21^

### Setting

An acute NHS healthcare trust in the Midlands that participated in Phase 1 of the implementation of Martha’s Rule. The healthcare trust is located in a city with an ethnically and culturally diverse population.

### Participants and recruitment

We used a purposive sampling strategy, seeking adults aged over 18 years who identified as being from diverse cultural and ethnic backgrounds and who had recent experience (within the past 5 years) of being an inpatient or as a relative/friend of an inpatient at the healthcare trust. We recruited participants through gatekeepers from the community resilience team at the local authority and through advertising at events and/or in spaces dedicated to the communities we sought to interview. Information about the study was circulated by email or by leaflet and verbally at community centres, libraries, family hubs, charities and faith-based organisations as well as community celebration events and events held to celebrate Refugee Week. Proficiency in English was not a barrier to participation. We translated study leaflets and information into locally relevant languages and offered interpreters.

A formal sample size calculation was not undertaken. Instead, sample size was informed by the concept of information power, which suggests that the more relevant information participants hold in relation to the study aim, the fewer participants are required.^22^ Given the study’s focused aim, the specificity of the sample and the richness and depth of the interview data, the research team judged that sufficient information power had been achieved to address the study objectives.

### Data collection

Informed written consent was received from all participants. In person, telephone or video call (Teams) interviews were arranged, with evenings and weekends offered to maximise participation. All interviews were conducted by OCL. Interviews took place between June and September 2025. Professionally qualified interpreters were used for four interviews. Questions were asked in English then interpreted into the required language by the interpreter, and participants’ responses were translated into English during the interview. The interpreter was briefed on the study aims and asked to provide as close a translation as possible to participants’ original meaning. An interview guide was developed based on Levesque’s access to healthcare framework.^23^ This multidimensional framework combines the accessibility of health services (approachability, acceptability, availability and accommodation, affordability and appropriateness) with the corresponding abilities of populations to access healthcare (ability to perceive, ability to seek, ability to reach, ability to pay and ability to engage). The interview guide explored four areas: participants’ previous experiences of hospital care; perceptions of Martha’s Rule and its potential role in responding to patient deterioration; preferences regarding communication and accessibility of information; and barriers and enablers to engaging with Martha’s Rule. The full guide is provided in online supplemental file 1. As Martha’s Rule was not fully implemented at the healthcare trust during data collection, participants did not have direct experience of it, so interviews explored anticipated rather than experienced barriers and enablers. Each participant received a £25 gift voucher in recognition of their time.

### Data analysis

Interview recordings were transcribed verbatim and all identifiable information was removed. The research team checked the transcripts for accuracy against the recordings. Transcripts were not returned to participants. NVivo V.15 was used for the management and coding of the data. The study was grounded in a critical realist perspective, acknowledging an independent reality that is experienced and given meaning to by individuals. The framework approach was used to organise and manage the data.^24^ This approach comprises five steps: (1) data familiarisation, (2) framework construction, (3) indexing and sorting, (4) data display and summary and (5) mapping and interpretation. After familiarisation with the transcripts, OCL identified preliminary codes using a subset of nine interviews. ELW created summaries of each interview and produced an initial set of descriptive codes capturing surface-level meaning. EL-W and OCL discussed these initial insights, which were then integrated with themes identified from the literature and organised into preliminary framework categories and sub-categories. We undertook member-checking through two workshops attended by seven interview participants. During these workshops participants reviewed the emerging findings and confirmed that the themes accurately reflected their experiences. EL-W then completed indexing and sorting of full transcripts so that the full dataset was incorporated within the framework. This was summarised in a matrix with participants organised into rows and the framework categories and subcategories organised into columns. Finally, we mapped the categories and subcategories onto the Levesque framework dimensions. Interview findings are presented and supported by anonymised data extracts from participants.

### Reflexivity

Interviews were conducted by one female researcher (OCL) who has an MSc and is a trainee psychotherapist. She has 15 years’ research experience in mental health, healthcare and trauma and has worked in intervention-based roles with clinical teams in prisons and in the community including managing a community-based mental health service. The participants were not previously known to the interviewer. EL-W is experienced in research with vulnerable populations and has a research interest in health inequalities. RC is a consultant in public health with an interest in understanding and reducing health inequalities in the NHS. LL-D is a nurse and qualitative healthcare researcher. Over 25 years she has investigated topics interrelated to patient safety, patient empowerment and decision-making and recognition of patient deterioration, in the context of nursing in the NHS. All authors had experience of working with qualitative data.

### Patient and public involvement

Patients and the public were actively engaged in two key stages of the study. Participants were offered the opportunity to attend workshops to discuss data analysis and develop recommendations arising from the research. Patients and the public are involved in dissemination of the findings including production of an animated video of the findings and development of a community outreach strategy to disseminate information about Martha’s Rule to communities.

## Results

### Participant characteristics

22 people participated in 21 interviews (2 people were interviewed together). These interviews lasted 25–154 min (M=66 min). Participants spoke between one and five languages (M=2.2 languages per participant) and 15 different languages were represented within the group. Interviews were online (n=5), by telephone (n=15) or in-person (n=1). Participant characteristics are reported in table 1.

**Table 1:** Participant characteristics

	N (%)
Total participants	22
Gender	
Woman	19 (86.4)
Man	3 (13.6)
Age	
24 and under	1 (4.5)
25–34 years	5 (22.7)
35–44 years	7 (31.8)
45–54 years	3 (13.6)
55–64 years	3 (13.6)
65–74 years	2 (9.1)
75+ years	1 (4.5)
Ethnicity	
Any other White background	1 (4.5)
Arab	3 (13.6)
Asian or Asian British	3 (13.6)
Bangladeshi	1 (4.5)
Black African	6 (27.3)
Indian	7 (31.8)
Pakistani	1 (4.5)
Education*	
No qualifications	4 (18.2)
Level 1 (eg, GCSE grades D–G or 3–1)	1 (4.5)
Level 2 (eg, GCSE grades A*–C or 9–4)	2 (9.1)
Level 3 (eg, A Level, advanced apprenticeship)	2 (9.1)
Level 4 or above (higher apprenticeship, foundation degree and above)	8 (36.4)
Other (vocational or work-related qualifications, other qualifications achieved in England or Wales, qualifications achieved outside England or Wales (equivalent not stated or unknown)	5 (22.7)
Not reported/missing	1 (4.5)
Employment status	
Full-time	3 (13.6)
Part-time	3 (13.6)
Not in paid employment	9 (40.1)
On maternity leave	1 (4.5)
Other	5 (22.7)
Not reported/missing	1 (4.5)
Disability*	
No known impairment	10
Long-standing illness or health concern	7
Physical impairment	3
Mental health difficulty	1
Specific learning difficulty	1
Other impairment not listed	1
Not reported/missing	3
Languages spoken	
English	18
Gujarati	6
Arabic	5
Urdu	4
French	3
Hindi	3
Bengali	1
Creole	1
Dari	1
Ibo	1
Polish	1
Punjabi	1
Telugu	1
Tigrinya	1
Twi	1

* Participants could self-report more than one disability.

GCSE, General Certificate of Secondary Education.

### Overview of findings

The analysis of the interview data generated three key themes. These themes reflect three of Levesque *et al’s* five key healthcare access dimensions and their corresponding patient abilities (approachability/ability to perceive; acceptability/ability to seek; appropriateness/ability to engage) (figure 1). The other two dimensions (availability/ability to reach; affordability/ability to pay) were not identified in the data.

**Figure 1:**
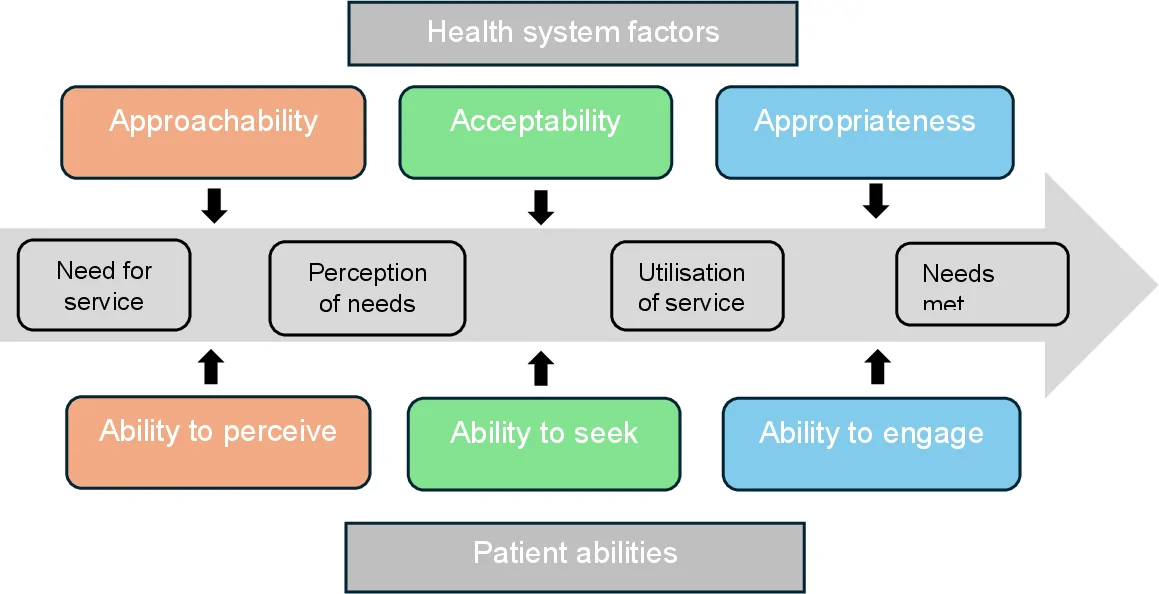
Conceptualisation of access to Martha’s Rule adapted from Leveque *et al*’s healthcare access framework.^23^

### ‘Approachability and ability to perceive’: visibility and accessibility of information about Martha’s Rule

A healthcare service is considered approachable when patients can identify that the service is available and know how to access it. Efforts to enhance a service’s visibility to its intended users involve being transparent, outreach work and provision of clear information and education about the services available which all influence a patient’s ability to identify a healthcare service.^23^ Our study participants highlighted that information provided about Martha’s Rule needs to be both visible and inclusive. They suggested that multiple channels should be used to share information about Martha’s Rule (as a patient service) including posters, leaflets, hospital TV screens, social media and providing information on admission.

You need to do more adverts for it, I don’t know, on social media, probably, will help … Everybody is using social media, so that will help more. GP reception, letters to households, texts, websites, and social media. (P04, Other ethnic group)

Outreach work with community and faith-based organisations (similar to existing health promotion programmes) was also advocated to reach those who may not come across information available at the hospital and to promote awareness raising via word of mouth.

That’s where people do go, like the mosque, that would be another place … somebody, if they’re are willing, even to have a little talk with the actual… in the places of worship where people do gather and explain what Martha’s Rule is about because that’s where we get all the people attending, especially when they have a lot of these health programmes, there are loads of health programmes at the [community venue] and places like that where we’re actually educated regarding the NHS, what is available …I think once a year I think they do it, all the doctors get together and they come and they take blood pressure, they talk about health problems, diabetes, et cetera, [Martha’s Rule is] something that could be actually introduced as well. (P14, Asian or Asian British)

Having a range of ways of sharing information about Martha’s Rule supports inclusion. Participants were aware that some formats could exclude certain groups. For example, older people in this population group were thought to be less likely to be able to engage with information available digitally and written information could exclude those who were not literate.

If you’re thinking of technology a lot of older people don’t use it still and there’s a lot of older people around who would rather pick up a leaflet when they’re at the hospital. (P19, Asian or Asian British)

Language was also an important factor in the inclusivity and accessibility of information about Martha’s Rule. Low English proficiency was identified by participants as increasing the likelihood of reduced awareness of the existence of Martha’s Rule.

Yeah, anybody who is not speaking English, anybody who’s new to this country, [they] won’t know [about] it. (P04, Other ethnic group)

Participants said that it was essential that information about Martha’s Rule was available in multiple languages, not just English.

If needed, I think, with the posters that they put up in different languages. … The best way would be something that maybe is in their language or something that’s like for them to see loud and clear, maybe something for them like in their language. … But for me, I think the idea would be the poster thing with the languages. (P11, Asian or Asian British)

It is also important that the language used to explain Martha’s Rule is clear and culturally sensitive. For example, using plain English and avoiding wording that suggests similarities with complaint procedures.

I think marketing it in a very, I guess, person friendly way, so to not make it seem like something scary, or like a complaint, because I feel like a lot of people - and if we’re looking at ethnic minorities who already have a lot of distrust in healthcare systems and formal authorities - labelling it as some kind of complaint procedure almost would probably put them off, because they’ll be like, “Oh, they’re going to ask me a load of questions. They’re going to ask this, they’re going to ask that,” and it can just be off putting because they just don’t want the hassle sometimes. (P06, Asian or Asian British)

To further support inclusivity, interviewees suggested that information about Martha’s Rule states universal eligibility which is particularly relevant for migrants and refugees.

[In relation to the covid vaccine] Because we were still waiting for the Home Office answer … and my partner, he was not registered with NHS … so he never went to the hospital or things like that. So when I read [the information about the covid vaccine] says regardless of your status, immigration, things like that I think, so then I book him with the GP and he got access to the vaccine. (P03, Asian or Asian British)

### ‘Acceptability and ability to seek’: language difficulties and perceived lack of agency as barriers to using Martha’s Rule

This theme concerns the social and cultural influences that shape patients’ willingness and capacity to seek and engage with a healthcare service such as Martha’s Rule.^23^ Language, as a key socio-cultural factor, plays a central role in shaping the healthcare experiences of those from diverse ethnic and cultural backgrounds. Language difficulties were perceived as a significant barrier to using Martha’s Rule. Additionally, patients’ personal beliefs and abilities, often rooted in their culture, can diminish agency in seeking healthcare, including using Martha’s Rule.

As well as impacting ability to access information about Martha’s Rule, low proficiency in English among some patients and families from diverse ethnic and cultural backgrounds was perceived as a significant barrier to using Martha’s Rule. Inadequate English proficiency was reported to be a cause of reluctance and a lack of confidence in raising or escalating concerns (such as patient deterioration) and asking for help. This was noted as a particular challenge for older generations.

I think it’s a language thing as well. Whereas I think I’m able to articulate my concerns a lot better, I suspect my Mum would feel out of her depth. (P01, Asian or Asian British)

Those who were less confident in speaking English could find it hard to clearly articulate their concerns about their own or a family member’s condition, resulting in barriers to using Martha’s Rule.

I think maybe they might think [accessing Martha’s Rule] it’s too complicated, you know. Or they don’t have the vocabulary …. or if they have an issue with getting their message across for whatever reason. Or, you know, they think they’re going to be misunderstood. (P19, Asian or Asian British)

Several participants explained that difficulties speaking English was a significant source of stress and anxiety when trying to explain concerns to healthcare staff and more generally when trying to access healthcare. A participant reported their mother-in-law’s experience of being in hospital:

So she was very stressed. It’s because she doesn’t speak in English. She was very stressed. She was really feeling uncomfortable because she couldn’t communicate. (P09, Other White background)

While translation services or digital devices were occasionally used to support communication with healthcare professionals, patients frequently relied on family members to interpret for them during healthcare interactions.

If I was not there [with my father-in-law in hospital], I don’t know, because the doctors keep asking questions and I was doing … the interpreter’s job, on behalf of my father-in-law … because they are asking him questions like what did you eat, and when did you eat, and what did you drink, and what is your habit for regular eating and what is your medication, and what is your NHS number. So I held all the details in my email, and I held the medication [details] … but the thing is I don’t know what [would have happened] if I was not there. (P10, Asian or Asian British)

To address language barriers to using Martha’s Rule, interview participants advocated for access to interpreters to support language needs.

Let’s say you are the one who’s picking up my phone, I’m calling you. I’m very stressed. I’m trying to explain in English and you’re trying to understand me, but it’s hard for you. So I think … you need interpreters. (P03, Asian or Asian British)

Some participants went further and suggested that there should be multilingual staff who engage with patients and families around Martha’s Rule, including, for example taking the calls when patients/families call the 24-hour Martha’s Rule phone line.

The benefits of providing interpreters for patients who are not proficient in English to support healthcare interactions were evident from examples given by participants, further reinforcing the need for the provision of interpreters in relation to Martha’s Rule.

She made me understand in my language that, look, [a] caesarean is nothing and I will try my best to do a normal delivery, but if you go for caesarean… Because I was scared of caesarean. Obviously, I don’t know anything. And she said, “No, look, this and this happens, after baby, you can walk, you can stand up in just 12 hours. You can have a shower, and you will be fine. Don’t worry, don’t worry.” So she makes me understand, in my language, very nicely. (P10, Asian or Asian British)

When communication does take place in English, participants suggested that it needs to be clear, use plain language and be at a steady pace as even those who know English can struggle with accents, fast speech and medical terminology.

There was a perception that those from ethnically and culturally diverse backgrounds often lacked confidence to raise concerns (about deterioration for example). Cultural norms were identified as being significant. For example, there is a cultural stigma around ‘bothering’ NHS staff or being a burden.

Because I’ve always found in our culture there’s always a sense of are we bothering people? There shouldn’t be that stigma. Are we bothering people too much? (P01, Asian or Asian British)

Participants described just accepting things, putting up with situations or feeling they should be grateful for basic things such as the provision of a meal in hospital rather than raising concerns or complaining.

Awareness of staff shortages and NHS pressures was also a barrier to raising concerns because of a reluctance to add to staff workload, which may be linked to cultural norms around not wanting to be a burden.

We see that the NHS is struggling. We see all these things, and sometimes a lot of people just want to be as less problematic as possible in their patient journey. (P06, Asian or Asian British)

Perceived differences in power between healthcare staff and patients and families also impact on willingness to raise concerns. Participants described feeling powerless and ‘at the mercy’ of healthcare staff, especially when there were cultural norms that reinforced deference to authority figures. There was a reluctance to raise concerns among some participants because they believed that their concerns would not be taken seriously.

Nobody will listen to you … they don’t want these concerns, they just fob you off, they’re really powerful these medical people. (P18, Asian or Asian British)

Social, cultural and demographic factors impacted some groups to a greater or lesser extent. Participants identified illegal migrants, refugees, older people and socially disadvantaged individuals as being least likely to raise concerns and use Martha’s Rule. Those who are educated, are familiar with the NHS or who have family to advocate for them are more likely to feel more empowered to raise their concerns.

### ‘Appropriateness and ability to engage’: reduced trust and confidence in healthcare and staff response can hinder engagement with Martha’s Rule

This dimension of Leveque’s framework relates to the appropriateness of the healthcare service in terms of its quality as well as patients’ ability to engage. An individual’s ability to engage in healthcare (eg, Martha’s Rule) is strongly determined by their capacity and motivation to participate which in turn is often influenced by the quality of interactions with healthcare services.

Many participants in the study discussed the quality of the healthcare that they or a family member had received in the past. In relation to staff behaviour towards them or a loved one, interviewees had mixed experiences. Many described instances where staff had been kind and caring, but many also reported experiences of staff being rude, unhelpful and in a few extreme cases, abusive.

But that time [having heart bypass] was really, really, really good. They had good manners. They were treating me nicely. (P02, Other ethnic group)

A small number of participants had experienced racism from healthcare professionals, including intragroup racism.

One female Muslim doctor and I think an Indian nurse saw me for an assessment. Now the Muslim doctor at no point did she meet my eyes. She was very offhand with me. When she asked the perfectly normal question, do you drink [alcohol], I said yes, and the look of disapproval on her face was staggering. I just thought I don’t feel like I’m in England anymore. I’m being judged and I’ve been raised in a mixed-race family so I was raised with alcohol being part of the culture, and it’s perfectly normal and acceptable but I felt judged by that woman. (P16, Asian or Asian British)

Lack of communication from healthcare professionals was also a common experience across interviewees.

My brother mentioned they were not really communicating. Like someone is not coming to speak to him, like explain things to him. (P08, Black, Black British, Caribbean or African)

Some participants had experienced being ignored, dismissed or not being believed when interacting with healthcare services.

I had to wait 18 hours, without investigation, without ECG, without anything, like to check my heart, and I had to wait that long of time. We’re waiting with my son in the room, and after that, the nurse, when I asked her, can someone come and check me, because I’m not feeling well at all, and my son, himself, he is not feeling well, he is diabetic … she told me you have to wait. (P02, Other ethnic group)

Overall, many participants reported poor experiences of care and this impacted negatively on participants’ confidence and trust in the NHS and its staff.

Participants highlighted the need for Martha’s Rule because of poor care experiences, particularly in circumstances when they had raised concerns but had been ignored or dismissed. Indeed, all participants who were asked (21 of 22 participants) felt that there was a need for Martha’s Rule.

Yeah, I think [Martha’s Rule is] a good idea because very often the family know a lot more about their loved one because they know what’s really going on, and then when you do mention it to them, they don’t listen, they think they know best, when you say oh, look, you’ve got a concern about this, and they just brush you off, they’re not really interested. (P18, Asian or Asian British)

Some participants felt that their previous experiences of poor care would make them more inclined to use Martha’s Rule.

I think I might be a bit more hasty or a bit more…. what’s the word? Likely to initiate it now rather than before my experience because I think before my experience, honestly, I would have been like, “Oh, it’s okay. They know what they’re doing.” I've always been that kind of person who’s like, “They know what they’re doing. They know what they’re doing.” I would be to my parents, “No, no, honestly, you’re just overreacting. It’s fine.” Whereas I think now I’m way more like, “Actually, you know what? They don’t always know what they’re doing.” Sometimes you do need to question things. (P06, Asian or Asian British)

However, other participants said that negative experiences actually undermined their confidence that Martha’s Rule could enhance patient safety and care.

And also like, you know, what would be the advice or what would happen with that call [to Martha’s Rule] or post that call? We don’t want to go back where nothing is backing that up in reality. So, you know, if we’re just having to then depend on the system as it is, we’re nowhere any better. So, this would be almost like an emergency call, [that would prompt action], well, is that really going to really happen? That would be my concern or would we still be taken from pillar to post sort of thing. (P19, Asian or Asian British)

Another important factor in the likelihood of using Martha’s Rule was the anticipated staff response. Some participants expressed concerns that the immediate care team might respond negatively to a patient or family member escalating concerns using Martha’s Rule and that this could lead to negative care interactions in the future.

But actually … it will create more hostility between staff and patients potentially. (P16, Asian or Asian British)

This would be a barrier to accessing Martha’s Rule for some, but others stated that they would disregard the risk of a possible negative response and go ahead and use Martha’s Rule anyway.

At that time I won’t even worry what people, what the staff think, because I think [about getting] the right treatment [for my relative], then – do you think I’m going to think about all these people? No. (P03, Asian or Asian British)

It was recognised that healthcare staff may perceive the use of Martha’s Rule by patients and families as questioning their care, but that they must accept it as a routine part of practice and should support patients and families to access Martha’s Rule if needed.

I suppose some [healthcare staff] might think, oh, that’s going to be a complaint about me but, you know, they shouldn’t really think like that. If they’re doing their job then that’s fine. They should encourage the patient or their carer or family or whatever, to say, you know, if you’ve got … any concern, if it’s something about us, what we’re doing on the ward, bring it to us [we can] follow it up and correct it or whatever. But if it’s anything any further they should then provide that information to say, look, this is a helpline [Martha’s Rule] this is where you need to go. (P19, Asian or Asian British)

## Discussion

To our knowledge, this is the first study to explore the perspectives of people from diverse ethnic and cultural backgrounds on the barriers and enablers to accessing Martha’s Rule. The findings highlight multiple potential barriers to accessing Martha’s Rule for this population at the patient and system level. Barriers related to how and where information about Martha’s Rule is communicated, socio-cultural factors such as language difficulties and perceived lack of agency, and reduced trust and confidence in healthcare arising from poor previous experiences of healthcare and anticipated negative responses from staff.

This study captured important perspectives by specifically focusing on participants from diverse ethnic and cultural backgrounds who have often been overlooked in the wider literature on patient/family activated escalation systems.^8^ We included those with limited or no English proficiency through the use of interpreters. Despite efforts to recruit men to the study, most of our participants (86%) were women. This is a limitation of our study, which may affect the generalisability of our results, though men in the sample did not report notably different views. We were unsuccessful in engaging significantly with the Polish community who form a substantial minority of the regional population, so their perspectives are under-represented in our data. Our study captured anticipated rather than experienced barriers to accessing Martha’s Rule, as it was not fully implemented at the point of data collection. A further limitation is that the study was conducted in a single acute NHS Trust in central England, and the findings may not reflect experiences in other NHS organisations or regions.

While Martha’s Rule has been recently implemented in England and research on its implementation is in its infancy, there is a small international evidence base on patient/family activated escalation systems. Studies have identified the need for multiple methods to raise awareness of patient/family-activated escalation systems to address low awareness as a barrier to access.^3 25–30^ Similarly, participants in our study suggested that posters, leaflets and information on TV screens on hospital sites, providing information on admission, sharing via social media and community outreach work should be used to raise awareness. Previous studies identified verbal explanation by clinical staff as the most essential component of information provision,^26 27 30^ as patients and families may struggle to process printed information when distressed,^30^ but participants in our study did not mention this preference, warranting further research into preferred modes of communication. Providing information in multiple languages as well as interpreters to support engagement with Martha’s Rule for those with limited or no English proficiency was a central theme in our study. Low proficiency in English can reduce confidence levels in raising concerns using patient/family activated escalation systems as patients and families may worry that they will not be understood. Previous research has identified both the lack of interpreting services as a barrier to accessing healthcare and the benefits for patient experiences and outcomes when professional interpreters are used.^31–35^ This is especially important given that around 1 million people in the UK cannot speak English well, or at all.^36^

Broadly, our study found that those from diverse ethnic and cultural backgrounds may lack confidence or feel they lack agency to raise concerns via Martha’s Rule. There were several factors underlying this. Cultural norms were identified as a barrier because of concerns about being a burden or ‘bothering’ staff, consistent with existing evidence.^5 18^ Reluctance to raise concerns also stemmed from feelings of powerlessness and an unwillingness to challenge perceived patient/healthcare professional hierarchies, which has been identified as an issue by other studies that included both minoritised and non-minoritised participants.^3 5 26 30 37 38^ These power imbalances can be amplified when patients/families are from diverse ethnic and cultural backgrounds or where there are language barriers. Previous studies with broader populations have identified that patients and families are concerned about imposing on already strained services if they escalate concerns^5 38^ and in this study, participants were acutely aware of staff shortages and NHS pressures, expressing reluctance to add to workloads. Lacking confidence or agency to raise concerns is present across many groups, not just those from diverse ethnic and cultural backgrounds.^8 39^

Previous poor experiences of care impacted negatively on participants’ confidence and trust in the NHS and its staff which can result in a reluctance to engage with healthcare, including patient safety initiatives like Martha’s Rule. This finding is consistent with wider evidence about experiences of healthcare among people from diverse ethnic and cultural backgrounds prompting disengagement and lack of trust.^18 40 41^ Apprehension that escalating concerns via Martha’s Rule would prompt a negative response from the current care team or negative repercussions on care was also a potential barrier to engaging with Martha’s Rule, consistent with previous research,^3 29 30 37 42^ although this was not a barrier for some participants in our study who would use Martha’s Rule despite perceiving this risk.

Barriers to accessing Martha’s Rule have the potential to exacerbate existing health inequalities for patients from diverse ethnic and cultural backgrounds^32^ with potentially greater impact on those affected by intersectional health inequalities such as women, older people and those who are socio-economically disadvantaged within these populations.^43^ Based on the findings of this study, we recommend several actions to support equitable implementation of Martha’s Rule and minimise the risk of widening existing health inequalities. Barriers to access can be addressed by ensuring information about Martha’s Rule is available in multiple languages and formats (digital and non-digital) and circulated widely, including through community outreach, increasing staff awareness that cultural norms may discourage raising concerns, supporting a culture of speaking up, routinely asking patients and families if they have any concerns, and building patient trust in Martha’s Rule to address hesitancy due to distrust in healthcare. The experiences of racism and intragroup racism reported by a small number of participants suggest that implementation of Martha’s Rule should be accompanied by efforts to address unconscious bias and promote culturally sensitive communication among all healthcare professionals, irrespective of their cultural background. Future research should investigate the extent to which those from diverse ethnic and cultural backgrounds access Martha’s Rule once it is fully implemented.

## Conclusion

This study has highlighted potential barriers to accessing Martha’s Rule for people from diverse ethnic and cultural backgrounds. Efforts to enhance the inclusivity of Martha’s Rule should focus on ensuring visibility and accessibility of information, increasing staff knowledge around barriers to access that stem from cultural norms and building patient/family trust in Martha’s Rule.

## Supplementary material

10.1136/bmjopen-2026-123741 online supplemental file 1

## Data Availability

Data are available upon reasonable request.
