# Supplementary material for: Exploring anticipated barriers to engagement with Martha’s Rule, a patient/family-activated escalation system to support early detection of patient deterioration, among ethnically and culturally diverse groups: a qualitative study
Source: BMJ Open. 2026 Nov 9;16(11):e123741. doi: 10.1136/bmjopen-2026-123741 (PMC13756373; doi:10.1136/bmjopen-2026-123741)
Supplement: Supplementary file 1 [file bmjopen-2026-123741.reviewer_comments.pdf]

PEER REVIEW HISTORY

BMJ Open publishes all reviews undertaken for accepted manuscripts. Reviewers are asked to complete a checklist review form and are provided with free text boxes to elaborate on their assessment. These free text comments are reproduced below.

ARTICLE DETAILS

Title (Provisional)

Exploring anticipated barriers to engagement with Martha's Rule, a patient/family-activated escalation system to support early detection of patient deterioration, among ethnically and culturally diverse groups: a qualitative study

Authors

Lutman-White, Eleanor; Ladeinde, Olufunmilola Christiana; Lees-Deutsch, Liz; Chapman, Rachel

---

VERSION 1 - REVIEW

**Reviewer**	**1**
**Name**	**Low, Joseph**
**Affiliation**	**Health Innovation Network, Insights**
**Date**	**08-Aug-2026**
**COI**	**NA**

This manuscript aims to explore the barriers faced by those from by those from diverse ethnic and cultural backgrounds in engaging with Martha's Rule – a patient/family activated escalation system to support early detection of patient deterioration. Qualitative interviews was used to explore community members based at one community hospital.

The manuscript is well written and clear but needs some additional additions and refinements before it is acceptable for publication.

Background:

Clear, but reference needed for Martha's rule (p3, l.12)

Methods

Generally well presented. No major issues but some points to address.

Methods: Study design. (p3, ll.53-54)

Incorporate this in the main body of the methodology text, not here. Reading through the methodology, it is clear that the authors have been following the guidelines. This section should focus on the study design used.

Methods: Data collection (p4, ll.25-26).

Please add what topics were discussed in the interview. Can you put the topic guide as an appendix? This will allow readers to see whether the authors are answering their research questions and if the findings presented in the results were generated by the interviews.

Methods: Data analysis

p4, l.39: Can the authors elaborate on how they dealt with the translated script? Did they only use the English translation of the 4 interviews not conducted in English or transcribed the interview in the original language of the participants. Please can they elaborate and note the limitations with the translated text. Ideally a back forward translation should have been performed but understandable why it wasn't done dur to time, costs etc.

p4, ll.53-56: Member-checking: Can the authors elaborate on this? Did they ask the same participants who they interview to attend the two workshops?

Results:

General comments: In qualitative methodology, it is important to get a sense of both what the of the majority of participants felt about a topic, but also whether a minority of participants disagreed. For example , did the males or the other white groups have any differences with the other groups?

Participant characteristics: Do the authors have any data of how many of the participants were born in the UK or their level of fluency in English?

P5, l.36: What does M=2.2 represent? Are they trying to say that most participants spoke at least 2 languages?

Table 1 (p5. Ll48-50): Under Gender, use female and males. Can the authors explain

Table 2: Can the authors explain the rationale for having this table with these quotes, which seems to be replicating what is in the text underneath. Usually, authors tend to put quotes in tables to save on word space, but as the authors have a wealth of quotes presented, I'm not sure of the purpose of this table eg. Sharing through multiple channels.

Would it be better to introduce the different sub-themes at the beginning of each theme and then sub-divide the sections by sub-themes.

Use of quotes:

i) In the interest of reducing the word count and reducing reader fatigue, can the authors use one substantial quote to support their key findings. If another quote is used, can the authors highlight how the second quote presented is different from the first quote, with detailed description of how it differs.

ii) Can the authors put some other broad demographics figure next to the ID identifier e.g. broad ONS ethnic categories or gender, as this will help readers understand who produced the data.

The quote on p13, ll.22-4 “A small number of participants…. Intragroup racism.” Is a very interesting finding was the incidence of racism experienced from health care professional including intragroup racism. This is quite nuanced and important to highlight as it is important that all health care professionals, irrespectively of their cultural background, should be challenging their own prejudices. Can the authors expand on this? This participant perceived that the doctor was using her religious belief in criticising the participant’s lifestyle. A key recommendation in implementing Martha’s rule would be to make all healthcare professionals, irrespective of their cultural background, to challenge their own internal bias.

Discussion:

Whilst this section does give a brief summary of the main findings with its limitations, can the authors highlight the main recommendations more clearly and succinctly in how Martha’s rule can be implemented using these preliminary findings.

Is there a recommendation needed in addressing racism (p15, l55 -60)? Whilst the authors correctly identify the issue of racism and the power imbalance between health professionals and patients, what recommendations can be recommended to improve the situation?

Is another key limitation the fact that the data comes from one acute NHS trust in the central England? Would the same results be found in other areas of England with a diverse population? .

Other future research topic could include exploring the feasibility and acceptabililty of delivering Matha’s rule from the perspectives of both NHS staff and patients and also establishing a key KPI of what Martha’s rule is trying to achieve and evaluating its impact.

Specific comments:

P15, ll. 16-17. This is a good point, but there appears to be little evidence of their actual voice being represented in the quotes in the results. Can the authors make these quotes more transparent in the result section?

VERSION 1 - AUTHOR RESPONSE

**Reviewer comment**	**Response**
Background:<br>Clear, but reference needed for Martha’s rule (p3, l.12)	A reference for Martha’s Rule has been added.

Methods: Study design. (p3, ll.53-54)<br><br>Incorporate this in the main body of the methodology text, not here. Reading through the methodology, it is clear that the authors have been following the guidelines. This section should focus on the study design used.	We have retained the reference to the COREQ checklist within the study design section as this seems to be the common location for it after reviewing other BMJ Open articles. We have slightly adjusted its location within the study design section.
Methods: Data collection (p4, ll.25-26).<br><br>Please add what topics were discussed in the interview. Can you put the topic guide as an appendix? This will allow readers to see whether the authors are answering their research questions and if the findings presented in the results were generated by the interviews.	The interview guide has been provided as an online supplemental file. We have provided further detail about the topics that were discussed in the interview in the data collection section.
Methods: Data analysis<br><br>p4, l.39: Can the authors elaborate on how they dealt with the translated script? Did they only use the English translation of the 4 interviews not conducted in English or transcribed the interview in the original language of the participants. Please can they elaborate and note the limitations with the translated text. Ideally a back forward translation should have been performed but understandable why it wasn't done dur to time, costs etc.	We used professionally qualified interpreters who adhere to a strict code of conduct regarding accuracy. Therefore, we did not consider a back-forward translation necessary. We have now explained in the text that interpreters were professionally qualified and that the interviews were interpreter-mediated where responses were translated orally during the interview rather than translation of an interview transcript after the interview.
p4, ll.53-56: Member-checking: Can the authors elaborate on this? Did they ask the same participants who they interview to attend the two workshops?	We have added to the text that a small number of participants who we interviewed attended the two workshops.
Results:<br><br>General comments: In qualitative methodology, it is important to get a sense of both what the of the majority of participants felt about a topic, but also whether a minority of participants disagreed. For example , did the males or the other white groups have any differences with the other groups?	Thank you for this comment. Exploring differences in experiences by gender and ethnic group was not an objective of the study, and the study was not designed or sampled to support meaningful comparisons between demographic groups. Our analysis focused on identifying patterns and themes across participants' experiences, although we did remain attentive to differences in views and we have identified such instances in the text, for example in relation to the quality of care received in the past and whether previous negative experiences of care would make participants more or less inclined to use Martha's Rule.
Participant characteristics: Do the authors have any data of how many of the participants were born in the	We did not ask participants where they were born or their level of fluency in English so are unable to report

UK or their level of fluency in English?	this.
P5, l.36: What does M=2.2 represent? Are they trying to say that most participants spoke at least 2 languages?	We intended "M = 2.2" to indicate that the mean number of languages spoken per participant was 2.2. We have revised the text to make this clearer.
Table 1 (p5. Ll48-50): Under Gender, use female and males. Can the authors explain	Sex refers to biological characteristics (e.g., female, male, intersex) and gender refers to socially and culturally constructed identities, roles, and expressions (e.g., woman, man). We asked participants about their gender identity rather than biological sex, and therefore we need to remain consistent to the terms we have used, displayed in our table.
Table 2: Can the authors explain the rationale for having this table with these quotes, which seems to be replicating what is in the text underneath. Usually, authors tend to put quotes in tables to save on word space, but as the authors have a wealth of quotes presented, I'm not sure of the purpose of this table eg. Sharing through multiple channels.	Thank you for this observation. We agree that the table largely duplicated information presented in the accompanying text and did not add sufficient additional value. In response to the reviewer's suggestion, we have removed the table.
Would it be better to introduce the different sub-themes at the beginning of each theme and then sub-divide the sections by sub-themes.	We have 3 main themes and no sub-themes so are unable to sub-divide the sections by sub-themes.
Use of quotes:<br><br>i) In the interest of reducing the word count and reducing reader fatigue, can the authors use one substantial quote to support their key findings. If another quote is used, can the authors highlight how the second quote presented is different from the first quote, with detailed description of how it differs.	We have removed additional quotes to ensure that one substantial quote is included to support the key findings.
ii) Can the authors put some other broad demographics figure next to the ID identifier e.g. broad ONS ethnic categories or gender, as this will help readers understand who produced the data.	As requested, we have added standardised ONS ethnic categories next to the direct quotations. To strictly maintain participant anonymity and prevent deductive disclosure in a qualitative sample, we have utilized the ONS's 5 broad high-level categories rather than detailed sub-groups.
The quote on p13, ll.22-4 "A small number of participants…. Intragroup racism." Is a very interesting finding was the incidence of racism experienced from health care professional including intragroup racism. This is quite nuanced and important to highlight as it is important that all health care professionals, irrespectively of their cultural background, should be challenging their own prejudices. Can the authors expand on this? This participant perceived that the doctor was using her	Thank you for this observation. We have added this reflection to the discussion.

religious belief in criticising the participant's lifestyle. A key recommendation in implementing Martha's rule would be to make all healthcare professionals, irrespective of their cultural background, to challenge their own internal bias.	
Discussion:<br><br>Whilst this section does give a brief summary of the main findings with its limitations, can the authors highlight the main recommendations more clearly and succinctly in how Martha's rule can be implemented using these preliminary findings.	We have amended the text to more clearly highlight that the recommendations for implementing Martha's Rule using these preliminary findings are at the end of the discussion section.
Is there a recommendation needed in addressing racism (p15, l55 -60)? Whilst the authors correctly identify the issue of racism and the power imbalance between health professionals and patients, what recommendations can be recommended to improve the situation?	We have now included a recommendation around addressing racism.
Is another key limitation the fact that the data comes from one acute NHS trust in the central England? Would the same results be found in other areas of England with a diverse population?	Thank you for this observation. We have added this limitation to the discussion.
Other future research topic could include exploring the feasibility and acceptabililty of delivering Matha's rule from the perspectives of both NHS staff and patients and also establishing a key KPI of what Martha's rule is trying to achieve and evaluating its impact.	This is an important future research topic and a large summative evaluation is currently underway which will offer a national understanding of the implementation of Marthas Rule, including how it has impacted staff and patients, how deterioration is managed in practice, what has helped and hindered its implementation, how it impacts inequalities, and its contribution towards fostering cultural change.
Specific comments:<br><br>P15, ll. 16-17. This is a good point, but there appears to be little evidence of their actual voice being represented in the quotes in the results. Can the authors make these quotes more transparent in the result section?	We are unable to respond to this point as we do not have access to specific line numbers.

VERSION 2 - REVIEW

**Reviewer**	**1**
**Name**	**Low, Joseph**
**Affiliation**	**Health Innovation Network, Insights**
**Date**	**13-Oct-2026**

**COI**	**NA**

I have read through the revised manuscript and the authors' comment, which I believe, have addressed both the reviewer and editors' comment. I am happy to accept this revised manuscript.
