# Supplementary material for: Exploring anticipated barriers to engagement with Martha’s Rule, a patient/family-activated escalation system to support early detection of patient deterioration, among ethnically and culturally diverse groups: a qualitative study
Source: BMJ Open. 2026 Nov 9;16(11):e123741. doi: 10.1136/bmjopen-2026-123741 (PMC13756373; doi:10.1136/bmjopen-2026-123741)
Supplement: Supplementary file 2 [file bmjopen-16-11-s001.docx]

**Interview Schedule**

**Exploring experiences of care in hospital**

We are interested in learning from you about any experiences you might have of being in hospital/a family member was in hospital and you were worried about how you were doing/your family member’s condition. We don’t mean situations where you came for an outpatient appointment but we mean situations where you/a family member were admitted to hospital. We only want you to talk about experiences you feel comfortable talking about. It’s fine if you’d prefer not to talk about experiences you’ve had.

Do you have any recent experiences of being in hospital/when a family member was in hospital you’d be willing to talk about? *(If participant does not have anything that they can/want to talk about, move to ‘Explain Martha’s Rule’)*

Could you tell me more about the worries you had about how you were doing/your family member’s condition, if you did have any worries?

Did you/a relative tell staff that you were worried about how you were doing/a family member’s condition?

If yes: Did staff listen to your worries?

Do you think staff shared your worries?

How were your worries responded to?

If you were not happy with the response, were you given other options?

Were you offered the option of a second opinion?

How would you have liked your worries to be responded to?

**Explain Martha’s Rule**

*Martha Mills died because she was not moved to intensive care quickly enough. Her family said to the team looking after her that they were worried about her condition getting worse, but their concerns were not listened to.*

*There have been cases similar to Martha’s where the concerns of the family and friends of hospitalised patients have not been responded to appropriately or on time. To improve care and make sure that the concerns of patients and their families are listened to, the NHS is implementing patient/family activated escalation systems across all hospitals in England (known as Martha’s Rule).*

*A patient/family activated escalation system gives patients and their families 24-hour access to a rapid review from an independent critical care team if they are worried about their or a family member’s condition in hospital and they have already raised concerns, but they do not feel they have been listened to. This would be in the form of a phone line that patients/family members could use.*

What do you think is good about Martha’s Rule?

What problems do you think there might be with Martha’s Rule?

What would you expect to happen if you called the phone line for Martha’s Rule? E.g. who would you expect to speak to? What would you want the response to be? How would you want the healthcare staff who answer the phone to respond? How would you want any other healthcare staff involved to respond?

Do you think Martha’s Rule is needed?

Who are the best people to notice changes in a patient’s condition?

When would you think using the Martha’s Rule phone line would be necessary?

Are there situations where you think using the Martha’s Rule phone line would be unnecessary or inappropriate?

How should the NHS describe Martha’s Rule and communicate about it? (Explore preferences for and understanding of terms ‘concern’ and ‘worry’).

What’s the best way to let people know about Martha’s Rule?

What kind of format would be best? E.g. posters, leaflets, multimedia, what languages?

Who should receive the information and when?

What are the important things to know about when communicating about Martha’s Rule?

Who would you speak to first if you had concerns about your own/a family member’s condition whilst in hospital?

**Barriers and enablers to engaging with Martha’s Rule**

What things would help people ask for a review of their/a family member’s care under Martha’s Rule (after having already raised concerns with the ward team which were not listened to)?

Are there any reasons why it would be difficult for people to ask for a review of their/a family member’s care under Martha’s Rule? What are they?

How could using Martha’s Rule be made easy for people?

*(Reiterate that the critical care team providing the independent review are separate from ward team)* How do you think the medical staff that were already providing care would respond to you/people raising your concerns via Martha’s Rule? Would this influence your/a person’s decision to ask for a review of care?

How do you think patients’ and family members’ relationships with ward staff might be after they had asked for a review of care under Martha’s Rule? (If negative views expressed: Do you think this would affect whether people would use Martha’s Rule?)

Which (groups of) people would be most likely to use Martha’s Rule?

Which (groups of) people would be least likely to use Martha’s Rule?

What would need to happen to help them use Martha’s Rule if they needed to?

How do you think people’s beliefs about health and healthcare might affect whether they decide to ask for a review of care under Martha’s Rule?

How do you think cultural, personal and spiritual beliefs might influence whether someone might use Martha’s Rule?

Do you feel you would have the confidence to raise concerns via Martha’s Rule?

Do you feel that you have trust in healthcare professionals and the healthcare system to raise concerns via Martha’s Rule?

Do you feel that you would be listened to if you raised concerns via Martha’s Rule?

**Final thoughts**

Overall, what are your thoughts about Martha’s Rule?

Do you have anything else you would like to add?
